# Inter‐parental conflict and empathy in early adolescence as predictors of dating violence perpetration in adolescence and adulthood

**DOI:** 10.1111/jora.12987

**Published:** 2024-05-30

**Authors:** Carlos N. Espinoza, Marlon Goering, Sylvie Mrug

**Affiliations:** ^1^ University of Alabama at Birmingham Birmingham Alabama USA

**Keywords:** dating violence perpetration, empathy, inter‐parental conflict, longitudinal

## Abstract

Witnessing inter‐parental conflict is associated with dating violence perpetration in late adolescence and adulthood. This relationship may be moderated by adolescents' empathy, with more empathic youth viewing their parents' conflict behavior as less acceptable, and thus refraining from dating violence perpetration. This study sought to determine if empathy buffers the effects of inter‐parental conflict in early adolescence on expected dating violence perpetration in early adolescence and actual dating violence perpetration in late adolescence and adulthood. Moreover, this study tested if expected dating violence perpetration in early adolescence and dating violence perpetration in late adolescence mediate the effects of inter‐parental conflict and empathy on dating violence perpetration in adulthood. Sex differences in these relationships were also examined. The sample included 412 adolescents (52% male, 73% Black, 25% White, 2% Other) who participated at three time points between 2006 and 2022 (T1–T3, *Mean* age = 13, 18, 28 years). Results indicated that higher inter‐parental conflict and lower empathy were associated with higher expected dating violence perpetration at T1 in both males and females. Inter‐parental conflict at T1 predicted higher dating violence perpetration at T3 through expected dating violence perpetration at T1 and dating violence perpetration at T2. Empathy at T1 predicted lower dating violence perpetration at T3 directly and also indirectly through lower expected dating violence at T1 and lower dating violence perpetration at T2 in both sexes. There were no interaction effects of inter‐parental conflict and empathy on expected or actual dating violence perpetration at any age.

## INTRODUCTION

Witnessing inter‐parental conflict in adolescence is associated with dating violence perpetration in both adolescence (Moretti et al., 2006) and adulthood (Cui et al., 2010; Ehrensaft et al., 2003). Less investigated are potential protective factors that may buffer the effects of inter‐parental conflict on later dating violence perpetration. One such protective factor may be empathy, which describes the ability to understand and feel others' pain as if it were one's own (De Kemp et al., 2007). Adolescents with higher empathy may view their parents' conflict behavior as a less acceptable way to treat a partner as they may empathize with the victim, and thus refrain from perpetrating violence towards their own dating partners in the future. Therefore, this study seeks to determine if empathy buffers the effects of witnessing inter‐parental conflict in early adolescence on expected dating violence perpetration in early adolescence, as well as actual dating violence perpetration in late adolescence and adulthood.

Furthermore, expectations of future dating violence perpetration in early adolescence may mediate the relationship between witnessing inter‐parental conflict and empathy in early adolescence and future dating violence perpetration (Windle & Mrug, 2009). Since dating violence perpetration during late adolescence has been linked with dating violence perpetration during adulthood (Greenman & Matsuda, 2016), links between inter‐parental conflict and empathy during early adolescence and dating violence perpetration in adulthood may also be mediated by dating violence perpetration during late adolescence. Given the evidence of sex differences in the links between inter‐parental conflict and both dating violence perpetration (Jouriles et al., 2012) and empathy (Van der Graaff et al., 2014), the effects of inter‐parental conflict and empathy on dating violence perpetration and the moderating role of empathy may vary by sex. Thus, this study also examines whether the effects of inter‐parental conflict and empathy in early adolescence on dating violence perpetration in adulthood are mediated by expectations of future dating violence in early adolescence and actual dating violence perpetration in late adolescence, as well as whether these relationships vary by sex.

### Ecological systems theory

The present study utilizes ecological systems theory (Bronfenbrenner, 1979) as a theoretical framework that informs this study's focus on empathy and expected dating violence perpetration (i.e., individual‐level factors), parental conflict behaviors (i.e., microsystem‐level factor), and the influence of inter‐parental conflict on behaviors towards a dating partner (i.e., meso‐system level factor). On the individual level, cognitive emotions such as empathy may serve as a buffer between witnessing inter‐parental conflict and (expected) dating violence perpetration, as understanding the negative effects of violence on others may disrupt the integration of harmful behaviors into one's internal working model and reduce the imitation of these behaviors. Additionally, according to the ecological systems theory, the microsystem involves factors in the child's immediate environment, such as the family unit, which influence a child's development. For example, witnessing inter‐parental conflict may impact children's internal working model of relationships with a significant other which then affects their behavior. When inter‐parental conflict witnessed within the family unit results in spillover effects that increase the risk for later imitation of these negative inter‐parental behaviors into dating relationships, a mesosystem is created (Bronfenbrenner, 1976).

### Dating violence

Dating violence is common, with 49% of women and 45% of men reporting having experienced psychological aggression victimization from a dating partner, and 42% of both women and men reporting physical dating violence victimization in their lifetime (Leemis et al., 2022). Among adults experiencing dating violence victimization, many report that their first time experiencing intimate partner violence occurred during adolescence (Centers for Disease Control and Prevention, 2022). Indeed, prospective research shows that experiencing dating violence in adolescence increases the likelihood of encountering it in adult relationships (Williams et al., 2008). Furthermore, dating violence victimization in adolescence is associated with other negative consequences, such as academic failure, substance abuse, and depression (Eaton et al., 2007; Johnson et al., 2014). Similarly, dating violence victimization in adulthood is associated with PTSD symptoms, poorer health, depressive symptoms, and substance use (Coker et al., 2002; Coker et al., 2005). Thus, efforts to reduce dating violence perpetration are important for minimizing negative effects on physical and mental health. Given that dating violence often begins in adolescence, understanding predictors of dating violence perpetration during adolescence and its continuation into adulthood are important.

Among key enabling factors for dating violence, as well as other forms of violence, are beliefs about the acceptability of violence. For example, attitudes supporting dating violence were associated with greater likelihood of dating violence perpetration in racial/ethnically diverse adolescents (Ali et al., 2011). Similarly, acceptance of dating violence predicted psychological teen dating violence perpetration among African American youth (Temple et al., 2016). Such normative beliefs about acceptability of violence may also contribute to adolescents' expected dating violence perpetration, defined as anticipated aggressive behaviors towards a future dating partner (Windle & Mrug, 2009). Expected dating violence perpetration may predict dating violence in a similar manner as beliefs and attitudes about violence. Indeed, expected dating violence perpetration in early adolescence is correlated with dating violence perpetration in late adolescence (Morris et al., 2015). Therefore, the present study includes expected dating violence in early adolescence both as an outcome and as a risk factor for later dating violence.

### Inter‐parental conflict and dating violence perpetration

In this study, inter‐parental conflict is defined as physical or verbal aggression between parents that is intending to cause harm when faced with a disagreement (Altenhofen et al., 2010). In a longitudinal study, 54–66% of adolescents reported witnessing psychological or emotional inter‐parental conflict (e.g., verbal abuse) in the past year while 11–26% reported witnessing physical parental conflict (e.g., assault) in the past year (El‐Sheikh et al., 2019). These findings indicate that many youth witness inter‐parental violence, which may provide a model for their future behavior towards dating partners.

Indeed, inter‐parental conflict has been associated with higher dating violence perpetration in several studies involving racial/ethnically diverse adolescents (Kinsfogel & Grych, 2004; Temple et al., 2013). Interestingly, both violent and nonviolent conflict behaviors between parents predict future dating violence perpetration in late adolescence (Tschann et al., 2009). Witnessing inter‐parental conflict in childhood and adolescence has also been linked to dating violence perpetration in adulthood. For example, witnessing inter‐parental conflict in early adolescence predicted dating violence perpetration in adulthood in a study consisting of mostly White youth (Cui et al., 2010). Additionally, witnessing inter‐parental conflict in middle adolescence was linked to dating violence perpetration in adulthood in racially/ethnically diverse adolescents (Ireland & Smith, 2009). Finally, adults who retroactively reported witnessing inter‐parental conflict before the age of 18 were more likely to report perpetration of physical dating violence than those who did not witness parental conflict in their youth (Plouffe et al., 2022). Thus, witnessing inter‐parental conflict in adolescence has been related to greater perpetration of dating violence across adolescence and adulthood in diverse youth populations. However, most of these studies have focused on middle and late adolescents, so less is known about the exposure to inter‐parental conflict in early adolescence and its relationship to expected dating violence in early adolescence, as well as actual dating violence perpetration in late adolescence and adulthood.

### Empathy and dating violence perpetration

One protective factor against the negative effects of witnessing inter‐parental conflict on dating violence may be empathy. Empathy is typically defined as the ability to understand and experience the feelings of others as if they were one's own (De Kemp et al., 2007). Empathy is thus thought to play an important role in social behavior, as understanding and experiencing the feelings of others should contribute to more prosocial and less antisocial behavior (Damon et al., 2006). Indeed, empirical research shows that middle adolescents with higher empathy levels are less likely to engage in abusive behavior such as dating violence 2 years later (McCloskey & Lichter, 2003). Similarly, lower empathy during middle school was linked to greater likelihood of perpetrating dating violence during high school in diverse youth (Espelage et al., 2020). However, few studies have examined longer‐term effects of empathy in early adolescence on dating violence perpetration in adulthood.

Interestingly, empathy levels fluctuate during early adolescence, suggesting this developmental period may be a critical time for empathy development (Van der Graaff et al., 2014). Interest in dating also begins in early adolescence, with 20% of 13–14 years old reporting having dated (Lenhart et al., 2015). As adolescents begin to date, higher empathy may improve the quality of these early dating relationships (Haugen et al., 2008). Positive experiences in adolescent dating relationships in turn predict higher quality of future dating relationships (Madsen & Collins, 2011). Thus, higher empathy in early adolescence may contribute to lower likelihood of dating violence from early adolescence to adulthood.

In addition to directly shaping the nature of dating relationships, empathy may also buffer the link between witnessing inter‐parental conflict and dating violence perpetration. Youth with higher empathy may empathize with the victim, view their parents' conflict behavior as less acceptable to resolve conflict, and prevent internalization of the observed violence in intimate relationships. However, little research has examined this moderating role of empathy. One study showed that empathy did not buffer the relationship between inter‐parental conflicts and dating violence perpetration in White and Hispanic youth (McCloskey & Lichter, 2003), but more research is needed. Thus, this study examines the buffering role of empathy in the links between inter‐parental conflict and dating violence perpetration across time in racially/ethnically diverse youth.

### Mediating role of expected dating violence

Given that witnessing inter‐parental conflict may lead to greater acceptance of dating violence perpetration (Ali et al., 2011) and that parental support of violence is mirrored by children (Martínez et al., 2021), it is possible that expected dating violence perpetration during early adolescence mediates the relationship between witnessing inter‐parental conflict during early adolescence and future dating violence perpetration. Previous cross‐sectional research has identified acceptance of dating violence as a mediator of links between inter‐parental conflict and dating violence perpetration in middle adolescence (Karlsson et al., 2016). However, no previous studies have examined expected dating violence as a mediator of inter‐parental conflict effects on future dating violence. It is also possible that expected dating violence mediates prospective links between lower empathy during early adolescence and future dating violence perpetration. Higher empathy has been associated with lower acceptance of violence during adolescence, which in turn has been linked with less involvement in later dating violence perpetration (Fernández‐González et al., 2019). Thus, this study examines expected dating violence perpetration during early adolescence as a mediator of dating violence perpetration in late adolescence and adulthood. Additionally, previous research has linked dating violence perpetration during late adolescence with subsequent dating violence perpetration in adulthood (Greenman & Matsuda, 2016), suggesting continued involvement in dating violence from adolescence to adulthood. Thus, this study also examines dating violence perpetration during late adolescence as a mediator of the effects of inter‐parental conflict and empathy during early adolescence on dating violence perpetration in adulthood.

### Sex differences

Research on sex differences in witnessing inter‐parental conflict and dating violence perpetration has yielded mixed results. Studies found that witnessing inter‐parental conflict in adolescence was associated with dating violence perpetration in males only (Kinsfogel & Grych, 2004), in females only (Jouriles et al., 2012), or in both males and females (Moretti et al., 2006). Research on dating violence perpetration in college students yielded similarly inconsistent results, with two studies suggesting witnessing inter‐parental conflict in adolescence predicted dating violence perpetration in both females and males (Gover et al., 2011; Hendy et al., 2003) and another study finding this relationship in females only (Baker & Stith, 2008).

Consistent sex differences have also been reported for empathy, with females having higher levels of empathy than males in both early adolescence and adulthood (Van der Graaff et al., 2014). Although no prior studies have investigated sex differences in the links between empathy and dating violence, there is some evidence that males exhibit stronger links between lower empathy and other types of aggressive behaviors than females, including bullying (Jolliffe & Farrington, 2011) and criminal offending (Jolliffe & Farrington, 2007). These differences are not surprising given that parents tend to be more likely to encourage the use of violence in males compared to females when faced with conflict and thus potentially promote a discrepancy between males and females in the occurrence of violent behavior (Farrell et al., 2011).

## CURRENT STUDY

**FIGURE 1:**
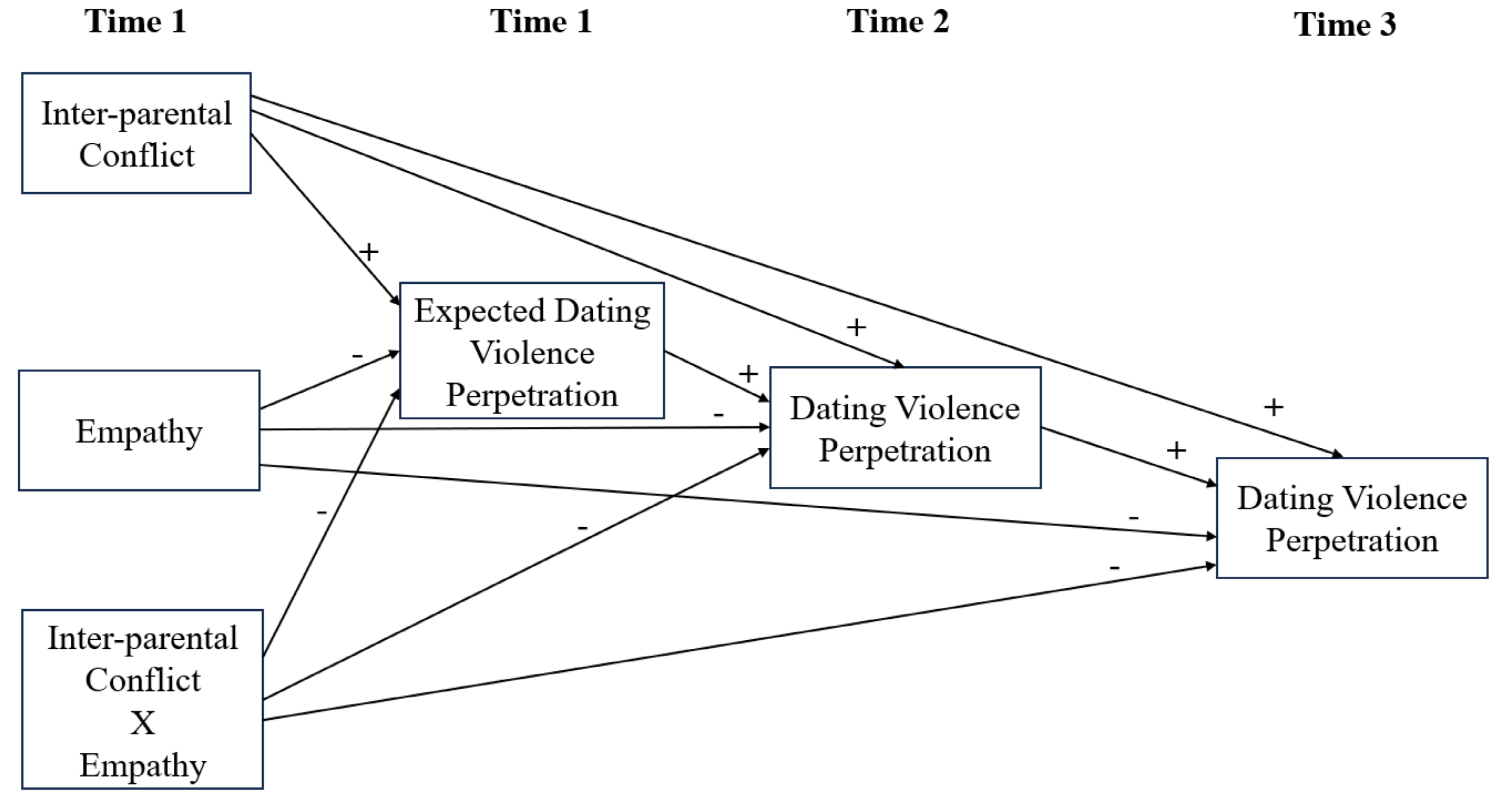
Hypothesized model. + = Hypothesized positive relationship, − = Hypothesized negative relationship. Sex differences in these relationships were also examined.

Extending previous research linking witnessing inter‐parental conflict, empathy, and dating violence perpetration, this study examines concurrent effects of inter‐parental conflict and empathy during early adolescence on expected dating violence, as well as their prospective effects on actual dating violence perpetration during late adolescence and adulthood and whether they are mediated by early expectations of dating violence and later actual dating violence (see Figure 1). This study also examines whether empathy can buffer the links between inter‐parental conflict and dating violence perpetration. It is hypothesized that both witnessing inter‐parental conflict and lower empathy in early adolescence will be associated with higher expected dating violence perpetration in early adolescence and higher dating violence perpetration in late adolescence and adulthood and that these effects would be mediated by expected dating violence perpetration in early adolescence and actual dating violence perpetration in late adolescence. Furthermore, it was hypothesized that empathy would buffer the effect of witnessing inter‐parental conflicts on expected dating violence perpetration and actual dating violence perpetration. Finally, given the equivocal findings on sex differences in links between inter‐parental conflict, empathy, and dating violence perpetration, no specific hypotheses regarding sex differences in these relationships could be made. Thus, sex differences are examined on an exploratory basis.

## METHODS

### Participants and procedures

This study includes 412 adolescents (52% male, 73% Black, 25% White, 2% Other) who took part in the Birmingham Youth Violence Study (BYVS) at three time points between 2006 and 2022. Initially, 704 adolescents were recruited from fifth‐grade classrooms of 17 public schools in a medium‐sized city in the Southeastern United States and achieved an 85% participation rate during recruitment. Participants were selected through a two‐stage probability sampling process with the goal of recruiting a representative sample of all students attending public schools in the surrounding area. This study only included participants that took part in the Wave 2 assessment. Given the present study's focus on the effects of inter‐parental conflict, only participants that lived in a household with at least two adults at Wave 2 were included in the analytic sample (*N* = 412). This report includes data from Wave 2 (referred to as Time 1; *N* = 412; mean age 13 years), Wave 3 (referred to as Time 2; *N* = 310; mean age = 18 years; 75% retention), and Wave 4 (referred to as Time 3; *N* = 250, mean age = 28 years; 61% retention).

At Time 1, adolescents participated together with their primary caregiver (72% Black, 27% White, 1% other; 84% Female; mean age = 39; 68% married). Family income ranged from below $5000 to over $90,000, with the median in the $30,000–$35,000 range. At Time 1, 65% of adolescent participants reported having dated and 87% reported having dated at Time 2. Participants were interviewed in private rooms at a university lab by trained interviewers who utilized Computer‐Assisted‐Self‐Interview (CASI) technology to assist participants in answering sensitive questions such as questions pertaining to dating violence perpetration. Participants were financially compensated for participating ($20 at Time 1, $50 at Time 2, $100 at Time 3). All procedures were approved by the Institutional Review Board.

### Measures

#### Inter‐parental conflict

At Time 1, adolescents reported on observed conflicts between their parents in the past 12 months using seven items from the Children's Perception of Interpersonal Conflict Scale and the Conflict Tactics Scale (Grych et al., 1992). If adolescents' parents did not live together, adolescents were asked to complete the questions thinking about other adults in their household or times when their parents lived together. The individual items addressed nonphysical violence (e.g., “When my parents have an argument, they say mean things to each other.”) as well as physical violence between parents (e.g., “My parents have pushed or shoved each other during an argument.”). Responses were made on a three‐point scale (0 – “False,” 1 – “Sort of True,” 2 – “True”). An exploratory factor analysis supported unidimensionality of the scale based on a scree plot. Scores were averaged with higher scores indicating more inter‐parental conflict (α = .76).

#### Empathy

At Time 1, adolescents reported on their empathy using the nine‐item Social–Emotional Competence Empathy subscale from the student version of the Social Skills Rating System (Demaray et al., 1995; Gresham & Elliot, 1990). The individual items addressed cognitive and affective empathy (e.g., “How often do you try to understand how your friends feel,” “How often do you feel sorry for others when bad things happen to them?”). The adolescents rated their responses to each item on a three‐point scale (0 – “Never,” 1 – “Sometimes,” 2 – “Very often”). An exploratory factor analysis supported unidimensionality of the scale based on a scree plot. Therefore, responses to all nine items were averaged with higher scores indicating more empathy (α = .71).

#### Expected dating violence perpetration

At Time 1, youth reported on their expected dating violence perpetration. A modified version of the Conflict in Adolescent Dating Relationships Inventory (CADRI) was used that began by asking participants “think about what you would do if you had a boyfriend or girlfriend and were angry with them” and were asked to respond to 17 items describing physical aggression (i.e., physical harm and threat of serious physical harm) and nonphysical aggression (i.e., emotional, relational, and verbal aggression) (Wolfe et al., 2001). Responses ranged from *Definitely would not* (1) to *Definitely would* (5). The correlation between physical and nonphysical dating violence was moderate (*r* = .58). Example items for physical aggression include “would hit, kick, or throw something at them,” “would you push shove or shake them,” whereas examples for nonphysical aggression include “do something to make them feel jealous” and “yell or scream at them.” The responses were averaged with higher scores indicating higher expected dating violence perpetration (α = .80).

#### Dating violence perpetration

At Times 2 and 3, participants reported their actual dating violence perpetration in the past 12 months using the 23‐item Conflict in Adolescent Dating Relationships Inventory (CADRI) describing physical, emotional, and verbal dating violence (Wolfe et al., 2001). At Time 2 participants were asked if they had ever dated before being asked about dating violence perpetration. If they endorsed not ever dating, they were not asked about dating violence perpetration, and this variable was coded as missing. These data were coded as missing instead of being excluded from analyses because individuals who did not report specifically dating someone at Time 2 may still have engaged in relationships such as “situationships” or “just talking” (Hardesty et al., 2024; Langlais et al., 2024) that would allow dating violence perpetration to take place. At Time 3, all participants were asked to think about their most significant romantic relationship in the past 12 months when answering dating violence perpetration questions.

Participants were asked to report on their behavior when they had a fight or argument with a romantic partner using a dichotomous scale (yes / no). They were asked questions such as “I slapped him/her or pulled his/her hair,” “I destroyed or threatened to destroy something he/she valued,” and “I brought up something bad that he/she had done in the past.” The correlation between physical violence and nonphysical violence at Time 2 and Time 3 were .57 and .46 respectively. Considering that nonphysical dating violence perpetration often co‐occurs with physical dating violence perpetration (Sears & Byers, 2010), physical and nonphysical dating violence perpetration were combined into a composite dating violence perpetration variable at Time 2 and Time 3. Constructing the variable in this manner also mirrors the inter‐parental conflict variable which included both physical and nonphysical inter‐parental conflict. The internal reliability of the scale was good at Time 2 (α = .85) and acceptable at Time 3 (α = .79).

#### Sociodemographic covariates

At Wave 1, adolescents reported on their biological sex and caregivers reported on the adolescents' date of birth. Participants' age was computed at each time point from date of birth and date of the interview. At Waves 1 and 2, caregivers also reported their annual family income before taxes using a 13‐point scale with response options ranging from “below $5000” to “above $90,000.” Wave 2 income was used, with missing responses substituted by family income at Wave 1 if present. Parent‐reported child's race at Wave 1 was coded as 1 for Black or another racial/ethnic minority and 0 for Hon‐Hispanic White.

### Analysis plan

Prior to the main analysis, a missing data analysis compared participants with any missing data to participants with complete data on all variables and covariates included in the analyses using independent samples t tests and chi‐square tests of independence. Descriptive statistics and bivariate correlations among all variables were also examined.

The first part of the main analysis consisted of a single hierarchical multivariate regression model conducted in Mplus 8.1. The model predicted expected dating violence perpetration at Time 1 and actual dating violence perpetration at Times 2 and 3 from covariates at Step 1, Time 1 inter‐parental conflict and empathy at Step 2, and the interaction of Time 1 inter‐parental conflict and empathy at Step 3. The Step 1 covariates included biological sex, racial/ethnic minority status, Time 1 family income, age at the outcome time point, and Time 1 expected dating violence perpetration when predicting dating violence perpetration at Times 2 and 3. The maximum likelihood estimator with robust standard errors (MLR) was used due to the nonnormal distribution of some of the variables. Missing data (13.5%) were handled with full information maximum likelihood (FIML) which preserves the full sample size (*N* = 412) and reduces bias under Missing at Random mechanism (Cham et al., 2017). All variables utilized in the analysis were specified as auxiliary variables to inform FIML.

The second part of the main analysis involved a mediation path model testing the indirect effects of inter‐parental conflict and empathy at Time 1 on dating violence perpetration at Time 2 through expected dating violence perpetration at Time 1 and on dating violence perpetration at Time 3 though expected dating violence perpetration at Time 1 and actual dating violence perpetration at Time 2. An indirect effect of the interaction effect would only be added if there were any interaction effects in the part 1 analysis. The indirect effects were tested with bias‐corrected bootstrapping using 5000 bootstrap samples.

In both parts of the main analyses, sex differences were tested with multigroup modeling. Likelihood‐ratio tests compared a model in which all paths were unconstrained to models in which one path of interest at a time was constrained to be equal by sex (Wang & Wang, 2019). Sex differences in the indirect effects were tested by comparing the fit of an unconstrained model to a model in which the individual paths included in the indirect effect were constrained to not differ by sex. Since the MLR estimator was used, the Satorra‐Bentler correction was performed when conducting the likelihood ratio tests (Satorra & Bentler, 2010). A significant likelihood‐ratio test indicated sex differences in the constrained path and the individual effects for males and females were interpreted from the unconstrained model.

Finally, sensitivity analyses were conducted to examine the robustness of the results if alternative analytic decisions were made. The first sensitivity analysis was conducted with the fully retained sample at Time 2 (*N* = 310) for Time 2 outcomes and with the fully retained sample at Time 3 (*N* = 250) for Time 3 outcomes. Another sensitivity analysis separated expected dating violence perpetration at Time 1 and actual dating violence perpetration at Time 2 and Time 3 into physical and nonphysical dating violence and examined the main and interactive effects of empathy and inter‐parental conflict together with sex differences for each type of dating violence separately.

## RESULTS

### Preliminary analyses

A missing data analysis showed that participants with missing data were more likely to report lower dating violence perpetration at Time 2 (*t*_(297)_ = −2.91, *p* = .004, Cohen's *d* = 0.34). Additionally, participants with missing data were more likely to be male (61% vs. 42%; χ^2^_(1)_ = 15.72, *p* < .001) and White (30% vs. 18%; χ^2^_(1)_ = 7.85, *p* = .005) compared to participants with complete data. However, participants with missing data did not differ from those with complete data in inter‐parental conflict, empathy, expected dating violence perpetration at Time 1, dating violence perpetration at Time 3, family income, or age at any time point.

Descriptive statistics and bivariate correlations among all variables are presented in Table 1. Higher empathy at Time 1 was associated with lower expected dating violence perpetration at Time 1, lower actual dating violence perpetration at Time 3, and lower inter‐parental conflict at Time 1. Witnessing inter‐parental conflict at Time 1 was associated with higher expected dating violence perpetration at Time 1 and higher dating violence perpetration at Time 2, but it was not related to dating violence perpetration at Time 3. Expected dating violence perpetration at Time 1 was associated with higher dating violence perpetration at both Time 2 and Time 3, and higher actual dating violence perpetration at Time 2 was associated with higher dating violence perpetration at Time 3.

**TABLE 1:** Descriptive statistics and correlations among all variables.

	M (SD)	1.	2.	3.	4.	5.	6.	7.	8.	9.	10.
1. Empathy T1	1.61 (0.29)	1.00									
2. Inter‐parental conflict T1	0.35 (0.38)	−.16**	1.00								
3. Expected dating violence T1	1.99 (0.55)	−.28**	.26**	1.00							
4. Dating violence perpetration T2	0.28 (0.20)	−.09	.18**	.35**	1.00						
5. Dating violence perpetration T3	0.24 (0.16)	−.20**	.09	.14*	.31**	1.00					
6. Age T1	13.13 (0.88)	−.14**	.16**	.24**	.11	.00	1.00				
7. Age T2	17.65 (1.04)	−.07	.09	.10	.17**	.07	.56**	1.00			
8. Age T3	27.74 (1.33)	−.19**	.07	.11	−.13	−.14*	.68**	.35**	1.00		
9. Female	48%	.13**	.11*	.25**	.30**	.09	−.01	.05	−.12	1.00	
10. Racial/Ethnic minority	75%	−.15**	.15**	.28**	.22**	.18**	.36**	.29**	.18**	.09	1.00
11. Family income	7.03 (4.09)	.09	−.17**	−.27**	−.17**	−.10	−.36**	−.24**	−.28**	−.12*	−.34**

*Note*: *p < .05, **p < .01.

### Main analysis

The results of the hierarchical multivariate regression model are presented in Table 2. At Step 1, the covariates explained 17% of variance in expected dating violence perpetration at Time 1, 21% of variance in dating violence perpetration at Time 2, and 7% of variance in dating violence perpetration at Time 3. At Step 2, the addition of inter‐parental conflict and empathy explained additional 8% of variance in expected dating violence perpetration at Time 1, less than 1% of additional variance in dating violence perpetration at Time 2, and additional 4% of variance in dating violence perpetration at Time 3. Higher inter‐parental conflict at Time 1 was uniquely associated with higher expected dating violence perpetration at Time 1 but did not predict dating violence perpetration at Time 2 or Time 3. Higher empathy at Time 1 was uniquely associated with lower expected dating violence at Time 1 and predicted lower dating violence perpetration at Time 3, but not at Time 2. At Step 3, the interactive effect of empathy and inter‐parental conflict did not improve the prediction of any outcome and was not significant at any time point. Multigroup analyses showed no sex differences in any of the main or interactive effects. Results from the multigroup analyses are reported in the supplementary materials (Table S1).

**TABLE 2:** Results from a hierarchical multivariate regression model predicting dating violence across time.

	Exp. dating violence perp. T1	Dating violence perpetration T2	Dating violence perpetration T3
	β	β	β
Step 1	*R* ^2^ = .17	*R* ^2^ = .21	*R* ^2^ = .07
Female	.22**	.23**	.01
Racial/Ethnic minority	.16**	.12*	.18**
Family income T1	−.14**	−.02	−.08
Age at outcome time point	.14**	.08	−.19**
Exp. Dating violence perp. T1	–	.24**	.08
Step 2	Δ*R* ^2^ = .08	Δ*R* ^2^ < .01	Δ*R* ^2^ = .04
Inter‐parental conflict T1	.14**	.07	−.01
Empathy T1	−.24**	−.02	−.20**
Step 3	Δ*R* ^2^ < .01	Δ*R* ^2^ < .01	Δ*R* ^2^ < .01
Inter‐parental Conflict × Empathy	.04	−.02	.03

*Note*: *p < .05, **p < .01.

Abbreviation: Exp., Expected.

### Indirect effects

**FIGURE 2:**
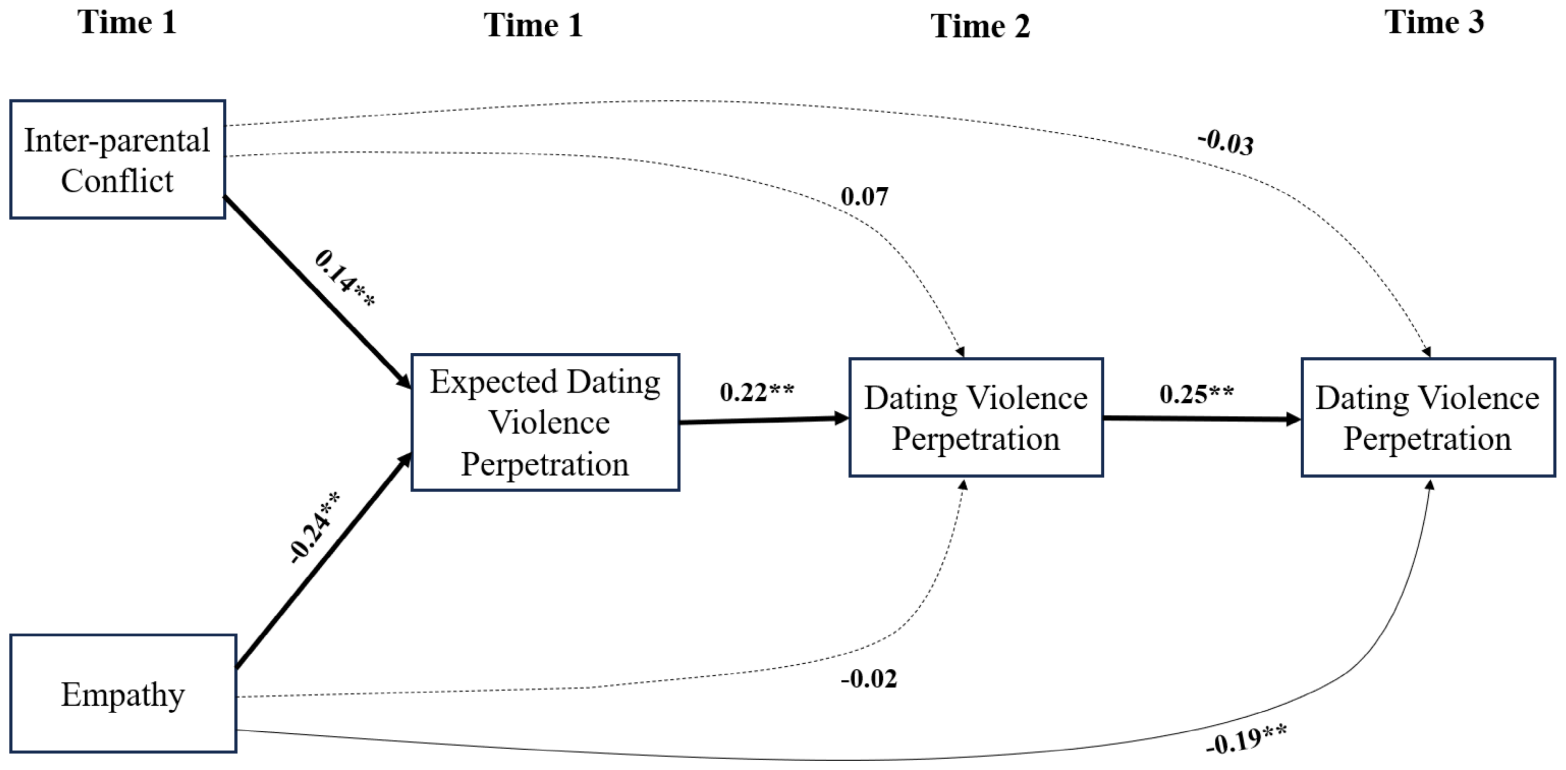
Mediation model results. Bold lines indicate mediation pathway. **p < .01.

The results from the mediation analyses are shown in Figure 2. The results showed that inter‐parental conflict at Time 1 predicts higher dating violence perpetration at Time 2 through higher expected dating violence perpetration at Time 1 (β = .030, CI_95%_ [0.009, 0.068]). Additionally, inter‐parental conflict predicted higher dating violence perpetration at Time 3 through higher expected dating violence perpetration at Time 1 and higher actual dating violence perpetration at Time 2 (β = .008, CI_95%_ [0.002, 0.021]). However, there was no indirect effect of inter‐parental conflict on dating violence perpetration at Time 3 through dating violence at Time 2 only (β = .016, CI_95%_ [−0.010, 0.056]). Similarly, the results showed that higher empathy at Time 1 predicted lower dating violence perpetration at Time 2 through lower expected dating violence perpetration at Time 1 (β = −.053, CI_95%_ [−0.097, −0.023]). Additionally, higher empathy at Time 1 predicted lower dating violence perpetration at Time 3 through lower expected dating violence perpetration at Time 1 and lower actual dating violence perpetration at Time 2 (β = −.013, CI_95%_ [−0.031, −0.004]) but not through dating violence perpetration at Time 2 only (β = −.005, CI_95%_ [−0.037, 0.022]). Direct, indirect, and total effects of the mediation analyses are reported in supplementary material (Table S2). Results from the multigroup analyses showed no sex differences in any of the indirect effects and can be found in supplementary material (Table S3).

### Sensitivity analyses

A sensitivity analysis which included only participants who returned at Time 2 (*N* = 310) replicated the original findings across main effects and the interaction between inter‐parental conflict and empathy at Time 1 in predicting dating violence perpetration at Time 2. Additionally, a sensitivity analysis involving only retained participants at Time 3 (*N* = 250) replicated the original findings across all main effects and the interaction between inter‐parental conflict and empathy at Time 1 in predicting dating violence perpetration at Time 3. These results confirm the robustness of utilizing Full Information Maximum Likelihood to handle missing data in the main analyses.

A sensitivity analysis conducted with expected physical dating violence perpetration at Time 1 as well as actual physical dating violence perpetration at Time 2 and Time 3 largely replicated the main results with two exceptions. Specifically, empathy at Time 1 did not predict reduced physical dating violence perpetration at Time 3 (β = −.12, *p* = .119). Additionally, the effect of inter‐parental conflict at Time 1 on physical dating violence perpetration at Time 3 differed by sex (Δχ^2^_(1)_ = 7.76, *p* = .015), with inter‐parental conflict predicting higher physical dating violence perpetration in males (β = .38, *p* = .004) but not in females (β = −.04, *p* = .657).

Similarly, the sensitivity analysis conducted with nonphysical expected dating violence perpetration at Time 1 and actual nonphysical dating violence perpetration at Time 2 and Time 3 replicated the main findings with two exceptions. Specifically, inter‐parental conflict at Time 1 did not predict expected nonphysical dating violence perpetration at Time 1 (β = .08, *p* = .100). Additionally, the effect of empathy at Time 1 on nonphysical expected dating violence perpetration at Time 1 differed by sex (Δχ^2^_(1)_ = 5.89, *p* = .014), with empathy predicting lower nonphysical expected dating violence perpetration in males (β = −.33, *p* < .001) but only marginally in females (β = −.12, *p* = .058). All other main effects, interactions, and sex differences replicated those found in the main analysis.

## DISCUSSION

The present study sought to explore the relationships between witnessing inter‐parental conflict and empathy with expected dating violence in early adolescence, as well as actual dating violence perpetration in late adolescence and adulthood. The present study also examined if empathy buffers any effects of inter‐parental conflict on concurrent expected dating violence perpetration in early adolescence and future dating violence perpetration. Additionally, expected dating violence perpetration in early adolescence and dating violence perpetration in late adolescence were examined as mediators of the effects of witnessing inter‐parental conflict and empathy on dating violence perpetration in adulthood. Finally, sex differences in these relationships were investigated.

### Inter‐parental conflict and dating violence

Witnessing inter‐parental conflict was uniquely associated with higher concurrent expected dating violence perpetration during early adolescence in both males and females. Thus, even though youth develop the ability to evaluate their parents' conflict behaviors more critically during early adolescence (Allen, 2008), they remain susceptible to the negative effects of witnessing poor conflict behavior in their home environment. The results revealed no direct effects of inter‐parental conflict on later dating violence perpetration in late adolescence and adulthood. However, witnessing inter‐parental conflict in early adolescence predicted later dating violence perpetration in adulthood indirectly through higher expected dating violence perpetration during early adolescence and dating violence perpetration in late adolescence in both males and females. Thus, witnessing inter‐parental conflict during early adolescence may put youth on a risk trajectory of higher expected dating violence perpetration in early adolescence which then leads to higher actual dating violence perpetration in late adolescence that persists into adulthood.

These findings suggest that witnessing conflict between parents sets up early expectations about normative or acceptable behavior in dating relationships by early adolescents, which then translate into actual behavior when dating in later adolescence and adulthood. These findings are in line with previous research suggesting that attitudes that are more accepting of violence mediate links between inter‐parental conflict and dating violence perpetration during adolescence (Temple et al., 2013). Results from the sensitivity analysis suggest that the effects of witnessing inter‐parental conflict on physical dating violence perpetration in adulthood significantly differed by sex, with witnessing inter‐parental conflict predicting increased physical dating violence perpetration in males only. These findings suggest that males are more likely to translate experiences of inter‐parental conflict into physical violence in dating relationships than females. These findings are in line with prior research that linked recalls of witnessing inter‐parental conflict before the age of 18 to physical dating violence perpetration in adulthood in males (Roberts et al., 2010) and a meta‐analysis finding that the link between witnessing interparental conflict in childhood and intimate partner violence perpetration in adulthood is stronger in males than females (Smith‐Marek et al., 2015). In sum, the present findings suggest that exposure to inter‐parental conflict during early adolescence is a risk factor for subsequent overall dating violence perpetration that extends into late adolescence and adulthood primarily through expected dating violence perpetration in early adolescence. Additionally, males are at an elevated risk for perpetrating physical dating violence when exposed to inter‐parental conflict in early adolescence.

### Empathy and dating violence

Higher empathy was associated with lower expectations of dating violence perpetration in early adolescence in both males and females. This result may be explained by greater concerns about the anticipated emotional harm to a dating partner in youth with higher levels of empathy. Previous research has suggested that empathy is associated with positive dating conflict resolution strategies such as compromising and perspective taking in youth (Courtain & Glowacz, 2019). Thus, youth with higher empathy may tend to utilize nonviolent strategies in response to conflict in future dating relationships.

Empathy in early adolescence predicted lower dating violence perpetration in adulthood directly as well as indirectly through lower expected dating violence perpetration in early adolescence, as well as lower actual dating violence perpetration in late adolescence in both males and females. These findings complement and extend prior reports of empathy predicting lower levels of dating violence perpetration during middle adolescence in males and females (McCloskey & Lichter, 2003). Importantly, the present findings point to enduring effects of early adolescent empathy on lower dating violence perpetration in adulthood, which may be explained by greater ability to cultivate healthier relationships with others (Boele et al., 2019). For example, empathy has been linked to higher relationship satisfaction (Ulloa et al., 2017), forgiveness (Fincham et al., 2002), and better emotion regulation (Schipper & Petermann, 2013), which may reduce the risk of dating violence perpetration. Interestingly, results from a sensitivity analysis suggest that the effect of empathy on concurrent nonviolent expected dating violence perpetration differed by sex, with empathy predicting lower nonviolent expected dating violence perpetration only in males. It has been argued that females are socialized to primarily utilize nonphysical violence by gender norms (Bowie, 2007) while male physical violence perpetration is encouraged by societal masculine stereotypes (Levant & Richmond, 2008). Indeed, early adolescent males tend to engage in more physical violence perpetration than females while females engage in more indirect violence perpetration than males (Österman et al., 1998). Thus, higher empathy in males may lead to lower expected nonphysical dating violence perpetration as this is a less commonly utilized form of aggression in males and not typically encouraged by gender norms.

### Inter‐parental conflict, empathy, and dating violence

Finally, the results did not support the hypothesized moderating role of empathy in the relationships between inter‐parental conflict and dating violence perpetration. This lack of moderation is consistent with previous results of no interaction between inter‐parental conflict and empathy on dating violence perpetration during middle adolescence (McCloskey & Lichter, 2003) and extend them to late adolescence and adulthood. Together, these results suggest that empathy in early adolescence is not able to buffer the effects of witnessing inter‐parental conflict on concurrent expectations of dating violence perpetration and future dating violence perpetration throughout adolescence and into adulthood. Instead, low empathy and exposure to inter‐parental conflict independently predict trajectories of dating violence perpetration. More research is needed to identify protective factors that buffer against the negative effects of witnessing inter‐parental conflict on dating violence perpetration.

### Implications

The current results suggest that increasing adolescents' empathy may be one way to reduce dating violence through adolescence and adulthood. Intervention research showed that empathy training utilized in anti‐bullying interventions has successfully reduced bullying behaviors (Garandeau et al., 2016; Şahin, 2012; Stanbury et al., 2009). Thus, empathy training may prove to be an effective component of interventions aimed at reducing dating violence in youth. Meta‐analyses of teen dating violence interventions suggest that existing school‐based programs mainly aim to reduce adolescent dating violence by targeting knowledge of dating violence and attitudes (De La Rue et al., 2017; Lee & Wong, 2020). Thus, future intervention studies should investigate whether including empathy training can reduce dating violence perpetration. Additionally, the present findings may inform early dating violence prevention programs in identifying at‐risk youth who are exposed to inter‐parental conflict in the home environment.

Additionally, future research should address the mechanisms through which low empathy and exposure to inter‐parental conflict in early adolescence contribute to the development of dating violence in later adolescence and adulthood. Bronfenbrenner's ecological systems theory may be one framework through which to further identify such mechanisms. For example, relevant mechanisms may include individual‐level factors such as communication and conflict resolution skills (Courtain & Glowacz, 2019), which may be diminished in youth who are exposed to microsystem‐level factors such as inter‐parental conflict and antisocial peers. Assessing exposure to inter‐parental conflict, empathy, these putative mediators, and dating violence involvement across multiple time points through adolescence and young adulthood would help elucidate the developmental processes through which empathy and parental conflict may lead to dating violence perpetration.

### Strengths and limitations

A key strength of this study was the longitudinal design that permitted the investigation of prospective effects of inter‐parental conflict and empathy on dating violence perpetration over time. Another strength was the inclusion of racial/ethnically diverse youth, who have been understudied in this line of research. Despite these strengths, the study was limited by reliance on self‐reports for all key variables, which may have introduced some bias and inflated associations among the variables due to shared method variance. Future studies should attempt to utilize multi‐source or multi‐informant reports. Additionally, there was some differential attrition with individuals who were White, male, and with lower dating violence perpetration being more likely to have missing data. Although the use of full information maximum likelihood reduced some bias due to this differential attrition, the results may be less generalizable to males, White individuals, and late adolescents with lower dating violence perpetration. The analyses examining expected dating violence perpetration as a mediator were limited by not examining expected dating violence perpetration at multiple time points, which did not enable the testing of temporal precedence in links between empathy and inter‐parental conflict with expected dating violence perpetration.

## CONCLUSION

In sum, the present study furthers our understanding of the relationships between inter‐parental conflict, empathy, and dating violence perpetration. The results indicate that witnessing inter‐parental conflict in early adolescence contributes to expected dating violence perpetration in early adolescence, which then translates into continued dating violence perpetration from late adolescence to adulthood. Additionally, empathy in early adolescence may contribute to lower expected dating violence perpetration during early adolescence as well as reduced dating violence in adulthood in both males and females. Moreover, empathy does not buffer the effects of witnessing interparental conflict on expected dating violence perpetration in adolescence or actual dating violence perpetration in late adolescence and adulthood. Together, these results suggest that reducing adolescents' exposure to inter‐parental conflict and nurturing empathy development during early adolescence may help reduce future perpetration of dating violence.

## CONFLICT OF INTEREST STATEMENT

All authors contributed in a manner that was deemed to warrant authorship of this manuscript. All participants consented to their participation in this study and all procedures were approved by the IRB. The authors declare that they have no conflict of interest.

## Supporting information


Data S1.

## Data Availability

The data that support the findings of this study are available from the corresponding author upon reasonable request.
