# Supplementary material for: Inter‐parental conflict and empathy in early adolescence as predictors of dating violence perpetration in adolescence and adulthood
Source: J Res Adolesc. 2024 May 30;34(4):1287–99. doi: 10.1111/jora.12987 (PMC11606263; doi:10.1111/jora.12987)
Supplement: Supplementary file 1 — Data S1. [file JORA-34-1287-s001.docx]

Table S1. *Results from the multi-group modeling to examine sex differences in the main and interaction effects.*

|  | Exp. Dating Violence Perp. T1 | Dating Violence Perp. T2 | Dating Violence Perp. T3 |
| --- | --- | --- | --- |
|  | Likelihood-ratio test: Δχ^2^(df), *p* | Likelihood-ratio test: Δχ^2^(df), *p* | Likelihood-ratio test: Δχ^2^(df), *p* |
| Step 2 |  |  |  |
| IP Conflict (T1) | 0.40 (1), .525 | 2.38 (1), .123 | 3.26 (1), .071 |
| Empathy (T1) | 1.95 (1), .163 | 0.01 (1), .911 | 0.00 (1), .958 |
| Step 3 |  |  |  |
| IP Conflict X Empathy | 1.74 (1), .187 | 1.45 (1), .229 | 0.00 (1), .995 |

*Note*. T1 – Time 1, T2 – Time 2, T3 – Time 3, IP – Inter-Parental.

Table S2

*Direct, Indirect, and Total Effects*

| Effects / Outcomes | Dating Violence T2  [95% CI] | Dating Violence T3  [95% CI] |
| --- | --- | --- |
| D: Inter-parental conflict T1-> Exp. Dating Violence T1 (Dating Violence T2) | [-0.055, 0.181] | [-0.168, 0.124] |
| I: Inter-parental conflict T1 -> Exp. Dating Violence T1 (Dating Violence T2) | **[0.009, 0.068]*** | **[0.002, 0.021]*** |
| T: Inter-parental conflict T1 -> Exp. Dating Violence T1 (Dating Violence T2) | [-0.028, 0.210] | [-0.143, 0.144] |
| I: Inter-parental conflict T1 -> Dating Violence T2 | **-** | [-0.010, 0.056] |
| D: Empathy T1 -> Exp. Dating Violence T1 (Dating Violence T2) | [-0.129, 0.099] | **[-0.316, -0.059]*** |
| I: Empathy T1 -> Exp. Dating Violence T1 (Dating Violence T2) | **[-0.097, -0.023]*** | **[-0.031, -0.004]*** |
| T: Empathy T1 -> Exp. Dating Violence T1 (Dating Violence T2) | [-0.182, 0.040] | **[-0.338, -0.070]*** |
| I: Empathy T1 -> Dating Violence T2 | - | [-0.037, 0.022] |

*Note:* D – Direct Effect, I – Indirect Effect, T – Total Effect; T1 – Time 1, T2 – Time 2, T3 – Time 3.

Table S3. *Results from the multi-group modeling to examine sex differences in the indirect effects.*

|  | Dating Violence Perp. T2 | Dating Violence Perp. T3 |
| --- | --- | --- |
|  | LR test: Δχ^2^(df), *p* | LR test: Δχ^2^(df), *p* |
| Inter-parental conflict T1 -> Exp. Dating Violence T1 | 3.71 (2), .156 | - |
| Inter-parental conflict T1 -> Dating Violence Perp T2 | - | 2.08 (2), .354 |
| Inter-parental conflict T1 -> Exp. Dating Violence T1 (Dating Violence Perp T2) | - | 5.17 (3), .159 |
| Empathy T1 -> Exp. Dating Violence Perp T1 | 5.40 (2), .067 | - |
| Empathy T1 -> Dating Violence Perp T2 | - | 3.76 (2), .153 |
| Empathy T1 -> Exp. Dating Violence T1 (Dating Violence Perp T2) | - | 6.87 (3), .076 |

*Note*. T1 – Time 1, T2 – Time 2, T3 – Time 3, Exp – Expected, Perp – perpetration, LR – Likelihood Ratio.
